# Effects of Specific Inhibitors for CaMK1D on a Primary Neuron Model for Alzheimer’s Disease

**DOI:** 10.3390/molecules26247669

**Published:** 2021-12-18

**Authors:** Paige Grant, Jitendra Kumar, Satyabrata Kar, Michael Overduin

**Affiliations:** 1 Department of Biochemistry, University of Alberta, Edmonton, AB T6G 2H7, Canada; pegrant@ualberta.ca (P.G.); kumar.jitendra@ualberta.ca (J.K.); 2 Centre for Prions and Protein Folding Diseases, Department of Medicine (Neurology), University of Alberta, Edmonton, AB T6G 2MB, Canada; skar@ualberta.ca

**Keywords:** Alzheimer’s disease, kinase inhibitor, CaMK1D, beta-amyloid, tau phosphorylation

## Abstract

Alzheimer’s disease (AD) is the most common cause of dementia worldwide. Despite extensive research and targeting of the main molecular components of the disease, beta-amyloid (Aβ) and tau, there are currently no treatments that alter the progression of the disease. Here, we examine the effects of two specific kinase inhibitors for calcium/calmodulin-dependent protein kinase type 1D (CaMK1D) on Aβ-mediated toxicity, using mouse primary cortical neurons. Tau hyperphosphorylation and cell death were used as AD indicators. These specific inhibitors were found to prevent Aβ induced tau hyperphosphorylation in culture, but were not able to protect cells from Aβ induced toxicity. While inhibitors were able to alter AD pathology in cell culture, they were insufficient to prevent cell death. With further research and development, these inhibitors could contribute to a multi-drug strategy to combat AD.

## 1. Introduction

Alzheimer’s disease (AD) is a progressive neurodegenerative condition which is the most common cause of dementia worldwide [1]. It is characterized by a slow decline in cognition including memory loss, which is the most common presentation, as well as poor judgement, difficulty communicating, and personality and behavioral changes [2]. There are two forms of AD, familial and sporadic, with sporadic being the most common. While familial AD is known to be caused by mutations in particular genes, the root cause of sporadic AD has remained more elusive. At the molecular level, AD is characterized most notably by neurofibrillary tangles and neuritic plaques, made up of aggregated tau and β amyloid (Aβ), respectively [3]. There are currently no effective treatments that successfully alter the progression of AD. While a multitude of studies have attempted to target Aβ and tau, none have been successful after clinical trials [4,5,6]. Conversely, in other diseases, kinase inhibitors have shown success [7]. Here, we explore the feasibility of using specific inhibitors for calcium/calmodulin-dependent protein kinase type 1D (CaMK1D) to prevent AD pathology in a cell culture model.

The most well-studied hypothesis regarding the root cause of sporadic AD is the amyloid cascade hypothesis [8,9]. In a non-amyloidogenic process, amyloid precursor protein (APP), a single pass integral membrane protein, is first cleaved by an α-secretase close to the membrane, then a γ-secretase on the intracellular side. This produces three benign fragments: the soluble sAPPα, p3, and amyloid intracellular domain (AICD). However, in an amyloidogenic process, APP is instead cleaved by a β-secretase, which cleaves APP in a location further from the membrane than the α-secretase, followed by the same γ-secretase cleavage. This produces sAPPβ, Aβ, and AICD [9,10]. The Aβ fragment forms oligomers and plaques in the extracellular space due to their high propensity for aggregation. The most common isoforms are 40 and 42 amino acids in length, with longer peptides such as Aβ_42_ being more toxic and aggregation prone [11]. In the simplest terms, the amyloid cascade hypothesis suggests that sporadic AD is caused by a sequence of events resulting directly from the action of these Aβ peptides, oligomers, and/or plaques. Another well-studied hypothesis regarding the root cause of AD is the tau hypothesis. In a healthy neuron, tau is a microtubule-associated-protein. It binds to tubulin and stabilizes microtubules, particularly in the axon [12]. In AD, tau undergoes abnormal post-translational modifications, the most well studied of these being hyperphosphorylation. When tau is hyperphosphorylated, it cannot bind tubulin. It falls off of microtubules, destabilizing them, preventing elongation and promoting their disassembly [5]. This loss of function alone is damaging for the cell, as microtubule functions are impaired and intracellular transport by kinesin and dynein are compromised. However, hyperphosphorylated tau also gains a toxic function. It aggregates in the cytoplasm of neurons into structures such as single straight filaments and paired helical filaments, which subsequently lead to the formation of neurofibrillary tangles (NFT) [12]. The tau hypothesis suggests that these tau aggregate structures are responsible for the degeneration of neurons and progression of AD. While over a dozen major tau kinases have been identified, few inhibitors have been tested clinically [13]. Tideglusib, an inhibitor for glycogen synthase kinase-3 (GSK3), is one of few exceptions, but this inhibitor failed to meet clinical endpoints in phase II clinical trials [14]. New targets are thus needed in this area.

The calcium hypothesis of AD suggests that disturbances in Ca^2+^ homeostasis are the driving factor of AD. Calcium ions (Ca^2+^) are important in neurons for neurotransmitter release, synaptic plasticity, gene expression, and many other cellular functions. Concentrations of Ca^2+^ are tightly controlled by various Ca^2+^ receptors, channels, pumps, antiporters, buffers and sensors, with each of these having an important role in maintaining Ca^2+^ homeostasis [15,16]. One of the most important effectors of Ca^2+^ function, calmodulin (CaM), is involved in numerous pathways in the cell including activation of CaM dependent protein kinases [17]. In AD, regulation of Ca^2+^ levels in the cell are disrupted, and thus, so are many of the downstream effects of Ca^2+^ stimulation [15,16].

One such downstream effect of disrupted Ca^2+^ levels in the brain may be aberrant activity of CaMK1D. Numerous studies have examined the role of *CaMK1D* in AD. A genomic convergence study first showed an association between single nucleotide polymorphisms (SNP) in *CaMK1D* and AD [18], and, subsequently, a genome-wide association study also identified *CaMK1D* as having SNPs associated with previously established AD SNPs [19]. Two studies on hydroxymethylation of *CaMK1D* have also shown associations with AD patients post-mortem [20,21]. One of these identified *CaMK1D* as one of only four genes with hydroxymethylation which was associated with clinical AD, as well as with both Aβ plaques and NFTs in post-mortem brain samples [20]. An examination of hydroxymethylation of DNA in blood samples from control and AD patients found alterations to *CaMK1D*, and suggested its use as a diagnostic marker for AD [22]. Despite the genetic and epigenetic evidence for CaMK1D’s involvement in AD, little research has been performed on CaMK1D in AD at the mRNA or protein level. In one study, *CaMK1D* mRNA levels in AD post-mortem basal forebrain samples were found to be downregulated by 75%, compared to normal aged controls. Similarly, protein levels in the AD basal forebrain were found to be downregulated by 63%, compared to normal aged controls. Interestingly, western blots for CaMK1D in post-mortem basal forebrain samples revealed unidentified bands at smaller sizes than full length CaMK1D, with one band approximately 15 kDa in size being unique to AD samples. The authors suggest that these may be CaMK1D fragments resulting from proteolysis, though this idea was not validated experimentally [23]. Additionally, CaMK1D is activated by CaMKK2 [24,25,26], and activates CREB [27], both of which are known to have aberrant activity in AD. Overall, it is clear that CaMK1D is a potential player in AD progression, although it remains under-studied.

**Figure 1:**
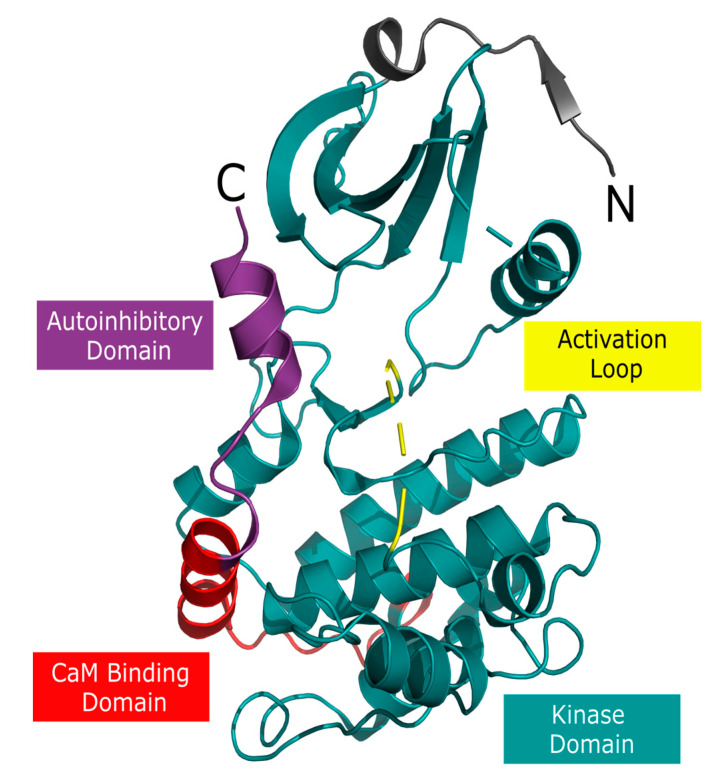
Crystal structure and domains of CaMK1D (PMID: 2JC6). The AID is shown in purple, CaM binding domain in red, activation loop in yellow, and the kinase domain in teal. Elements that do not fall into these categories are shown in grey. The termini are indicated by “N” and “C”.

The CaMK1D protein is a member of the CaMKI family of Ca^2+^/CaM dependent kinases, which consists of four isoforms (α, β, γ, δ) from separate genes. CaMK1D is expressed widely, and most highly in the brain [26,27]. Its domain structure is similar to that of other CaMKs, consisting of a kinase domain, a CaM binding domain (CBD), and an autoinhibitory domain (AID) that overlaps the CBD (Figure 1). In an inactive state, CaMK1D’s AID sits along the surface of the active site, blocking the enzymatic activity. Binding of CaM results in exposure of the active site as the AID relocates. This both allows a CaMKK to access the activation loop, phosphorylating and activating it at T180, as well as allows substrates to access the active site [26].

**Figure 2:**
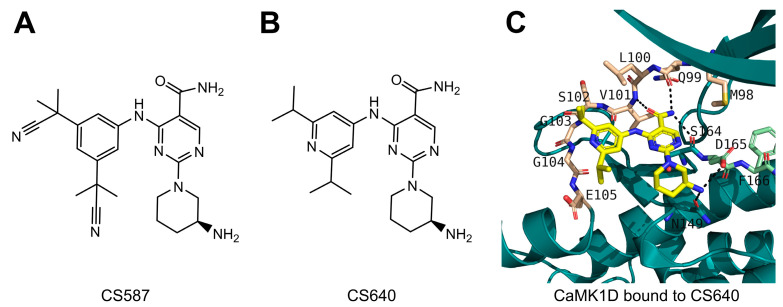
Structures and binding of CaMK1D inhibitors. (**A**) Structure of CS587. (**B**) Structure of CS640. (**C**) CS640 bound to CaMK1D in the ATP binding pocket. The P-loop has been made transparent for better visibility. Adapted from [28].

Specific inhibitors have been developed for CaMK1D, intended for in vitro and in vivo studies on diabetes and triple-negative breast cancer, and these compounds were able to improve insulin sensitivity in diet-induced obese mice [28]. Here, two inhibitors were used: CS587, and CS640 (Figure 2). Both of these are competitive inhibitors that engage in the ATP binding pocket and have IC_50_’s for CaMK1D in the nanomolar range at both the enzymatic and cellular levels. Both also show a strong selectivity for CaMK1D over related kinases; however, due to highly conserved active sites within the CaMKI family, these compounds have limited selectivity for CaMK1D over other CaMKI isoforms [28].

In this work, the effects of CaMK1D inhibitors on AD-like indicators in Aβ treated primary neurons were examined, taking into account the major AD hypotheses outlined above. Aβ_1–42_ oligomers were used to induce an AD-like state in mouse primary cortical neuron cultures, and tau phosphorylation was used as a marker of an AD-like state in these cells. The inhibitors were first tested for toxicity to primary neurons, and showed no significant toxicity up to 1 µM concentration. Subsequently, the effects of the inhibitors on tau phosphorylation and cell viability were explored. While cell viability did not appear to be affected by the presence of inhibitors at non-toxic concentrations, the phosphorylation of tau was reduced to near control levels.

## 2. Results

### 2.1. Neurotoxicity of CaMK1D Inhibitors

**Figure 3:**
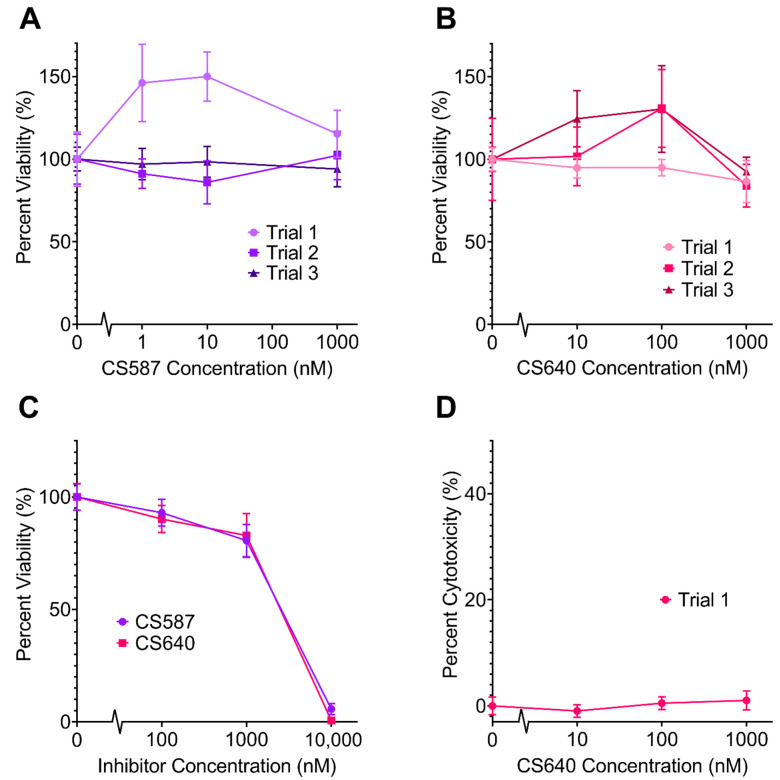
Effect of varying concentrations of CaMK1D inhibitors on primary neurons. Viability of mouse primary cortical neurons was measured by MTT assay. The response from cells treated with no CaMK1D inhibitors was normalized to 100% viability. Results of three separate MTT assay trials using (**A**) CS587 and (**B**) CS640. (**C**) Results of MTT assays using the inhibitors at higher concentrations, up to 10 µM. (**D**) Results of one LDH assay trial with CS640. Points represent the mean of six replicates, and error bars represent standard deviation.

The neuronal cell toxicities of CaMK1D inhibitors CS587 and CS640 were examined to determine their suitability for further studies in neurons and animals. In order for the compounds to be clinically useful, concentrations high enough to bind a significant portion of the CaMK1D population need to be achieved without compromising cell viability. CaMK1D inhibitors were tested for toxicity in mouse primary neuronal cell cultures by tetrazolium (MTT) colorimetric assays of cellular metabolic activity and lactate dehydrogenase (LDH) assays of cytotoxicity (Figure 3). In concentration ranges from 1 nM to 1 µM in the cell culture media, no significant loss of cell viability was observed from either inhibitor, and no significant increase in cytotoxicity was observed (Figure 3A,B,D). At concentrations of 10 µM, both inhibitors were found to be toxic by MTT assay. At this concentration, CS587 left less than 6% viability, and CS640 left less than 1% viability (Figure 3C). In one trial, CS587 seemed to increase the viability of these cells at concentrations of 1 nM and 10 nM by approximately 1.5 times (*p* = 0.0033 and *p* = 0.0003, respectively) (Figure 3A, Trial 1); however, this was not repeatable. From this data, it was determined that CaMK1D inhibitors could be used in further experiments with media concentrations up to 1 µM without detrimental effects on cell viability.

### 2.2. Effect of Inhibitors on Aβ Toxicity

**Figure 4:**
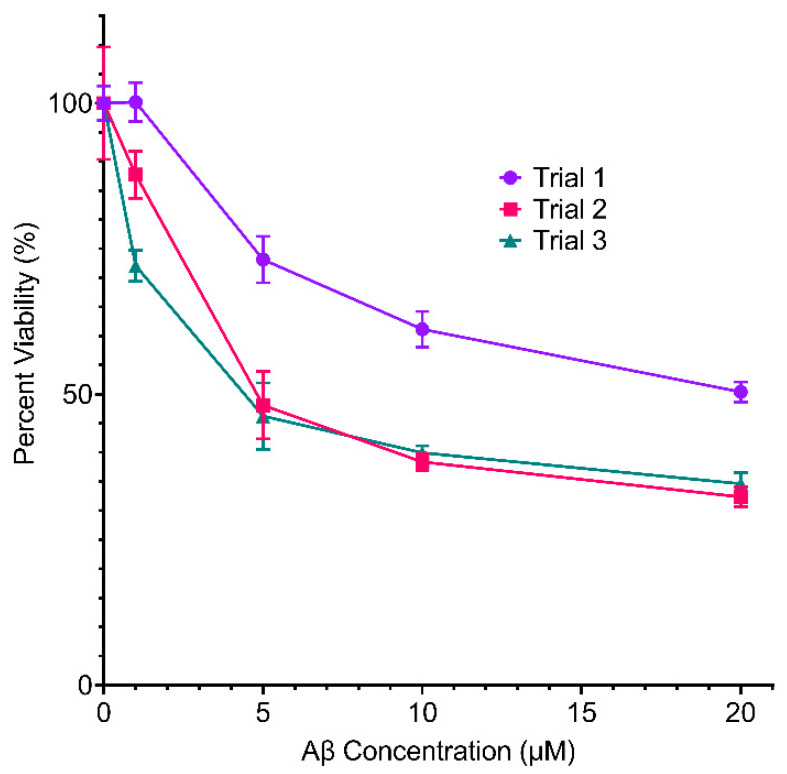
Effect of varying concentrations of Aβ_1–42_ on the viability of primary neurons. Viability of mouse primary cortical neurons was measured by MTT assay in three separate trials. Points represent the mean of six replicates, and error bars represent standard deviation.

**Figure 5:**
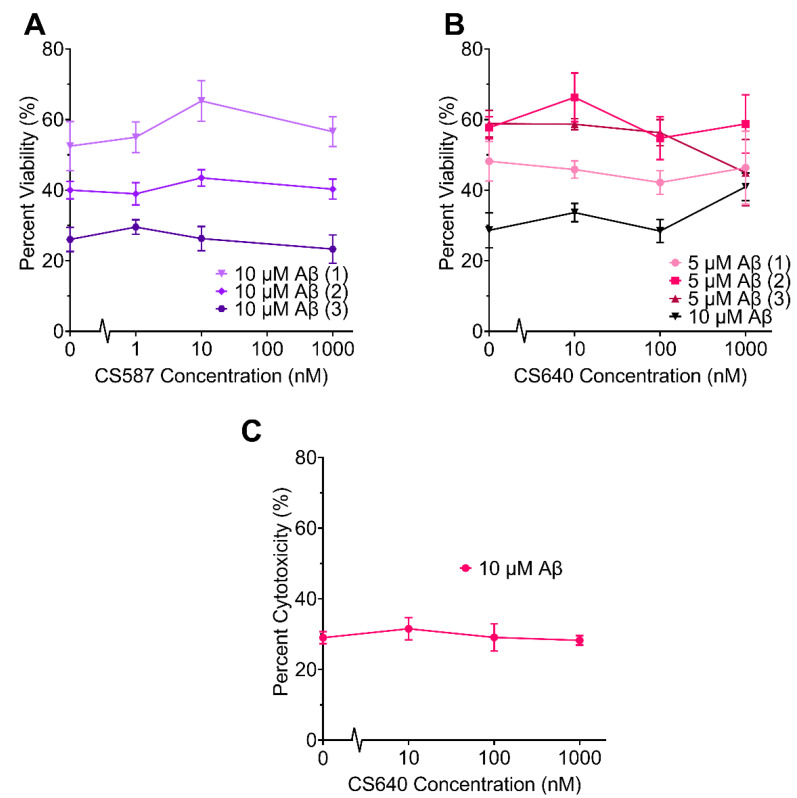
Effect of varying concentrations of CaMK1D inhibitors on the viability of primary neurons treated with Aβ_1–42_ oligomers. Viability of mouse primary cortical neurons was measured by MTT assay. The response from cells treated with no CaMK1D inhibitors or Aβ_1–42_ was normalized to 100% viability (shown in Figure 3). Trial numbers are indicated in parentheses. (**A**) Results of three separate MTT assay trials using CS587 and 10 µM *A*β_1–42_. (**B**) Results of three separate MTT assay trials using CS640 and 5 µM *A*β_1–42_, as well as one trial using 10 µM *A*β_1–42_. (**C**) Results of one LDH assay trial with CS640 and 10 µM *A*β_1–42_. Points represent the mean of six replicates, and error bars represent standard deviation.

In order to determine whether or not CaMK1D inhibitors altered the cellular sensitivity to toxic of Aβ oligomers, MTT assays and LDH assays were performed on mouse primary neurons treated with Aβ_1–42_. First, these assays were performed with varying concentrations of Aβ_1–42_, to determine an appropriate concentration to use in further assays (Figure 4). In the range of 5–10 µM Aβ_1–42_, there was a consistently statistically significant reduction in neuronal viability, so this concentration was used in further assays. These assays were performed in the presence and absence of varying concentrations of CS587 and CS640 (Figure 5). The CS587 compound was tested in the presence of 10 µM Aβ. In one trial, the viability of neurons treated with both 10 nM CS587 and 10 µM Aβ_1–42_ was higher than cells treated with 10 µM Aβ_1–42_ alone by about 1.24 times (*p* = 0.0064) (Figure 5A(1)). In all other trials, and at all other concentrations (1 nM to 1 µM), CS587 did not have a significant effect on neuronal viability in the presence of 10 µM Aβ_1–42_ (Figure 5A). The CS640 compound was tested in the presence of both 10 µM and 5 µM Aβ_1–42_. In one trial, the viability of neurons treated with both 1 µM CS640 and 10 µM Aβ_1–42_ was higher than neurons treated with 10 µM Aβ_1–42_ alone by approximately 1.4 times (*p* = 0.0009) (Figure 5B; 10 µM Aβ_1–42_), but, again, this could not be repeated. In all other trials, and at all other concentrations (10 nM to 1 µM), CS640 did not have a significant effect on neuronal viability in the presence of either 10 µM or 5 µM Aβ_1–42_ (Figure 5B). Overall, this data suggests that CaMK1D inhibitors are not able to consistently rescue mouse primary neurons from Aβ induced toxicity.

### 2.3. Effect of Inhibitors on Tau Phosphorylation

**Figure 6:**
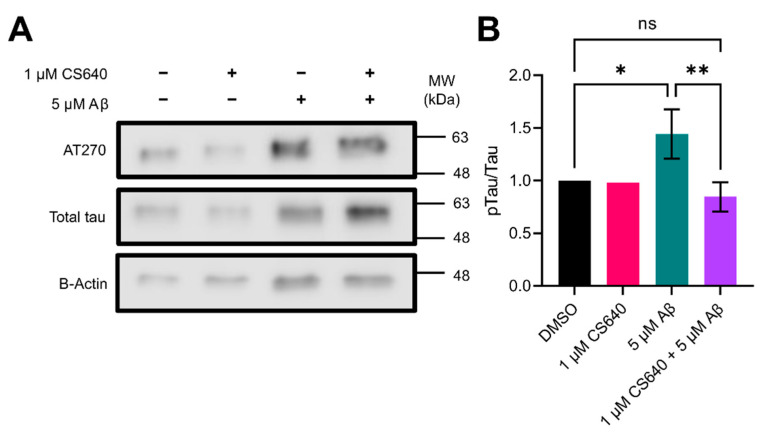
Relative abundance of phosphorylated tau in mouse primary neuronal cell cultures. (**A**) A representative western blot showing the relative abundance of tau phosphorylated at Thr181 (AT270), total tau, and β-Actin in the absence and presence of 1 µM CS640 and/or 5 µM Aβ_1–42_. (**B**) Relative abundance of tau phosphorylated at Thr181 (AT270) over total tau in the absence and presence of 1 µM CS640 and/or 5 µM Aβ_1–42_, calculated from quantifications of three replicates of western blots. Error bars represent standard deviation. Only one replicate of 1 µM CS640 alone was performed, and it is thus excluded from the statistical analysis. ns = not significant. ^ns^ *p* = 0.5004, * *p* = 0.0318, ** *p* = 0.0085.

Another AD related marker examined was phosphorylation of tau. Phosphorylation of tau at Thr181 in mouse cortical primary neuronal cultures was observed by western blotting using the AT270 antibody, and total tau was observed using the Tg5 antibody (Figure 6). Treatment with 1 µM CS640 alone had little to no effect on the phosphorylation level of tau at this site. Treatment with 5 µM Aβ_1–42_ caused an increase in tau phosphorylation at this site of 1.45 times (*p* = 0.0318). This increase was ablated by co-treatment with 1 µM CS640 (*p* = 0.0085). Here, the phosphorylation level of tau at this site decreased to about 0.85 times the DMSO control, statistically indistinguishable from the DMSO control (*p* = 0.5004). Thus, the CaMK1D inhibitor CS640 was able to revert tau phosphorylation at Thr181 to control levels.

## 3. Discussion

With little to no success in altering the progression of AD in clinical trials, it is prudent to explore further options in the field of AD research beyond the classic Aβ targeting [4]. Specific kinase inhibitors have proven to be effective cancer treatments, and protein kinase deregulation plays a role in virtually all major disease types, with many currently under development [7]. Having multiple kinases involved, AD is a clear candidate for further investigation of kinase inhibitors. CaMK1D, a kinase for which specific inhibitors have recently been developed, and which has been shown to be altered in AD, is such a candidate for this type of study.

Originally, there were concerns that these particular kinase inhibitors could potentially exhibit toxicity toward neuronal cells, even at low concentrations. CaMK1D is expressed most highly in the brain during early development, and is important for basal dendritic growth [29]; these inhibitors have nanomolar level IC_50_ values for CaMK1D, and the inhibitors have some off-target effects on other members of the CaMKI family [28]. Thus, it was reasonable to expect that they may have a detrimental effect on neuronal cells. However, this was not the case. At levels in media up to 1 µM, neither inhibitor showed any significant neurotoxicity. However, virtually all neurons were killed by 10 µM of either compound. These results can be used to inform dosage in future animal studies; dosages used previously remained below 10 µM in vivo, with the maximum concentration reaching 3 µM [28]. Naturally, a lack of neurotoxicity is an important attribute in a compound being used for research on a neurodegenerative disease such as AD. These inhibitors can be confidently used in further cell-based AD models, and researchers can proceed with more confidence in animal studies, whether focusing on breast cancer, diabetes, or AD, as neurotoxicity concerns have been addressed.

Increased tau phosphorylation is a clear AD indicator, and it is temporally correlated with brain structure changes in AD patients, as seen by MRI [30]. The particular phosphorylation site examined here, Thr181, is an early marker of AD which has been suggested as a cerebrospinal fluid diagnostic marker of AD [31], and has been previously shown to have increased phosphorylation induced by Aβ oligomer treatment. The increase in tau phosphorylation observed here is consistent with previous results [32], and Aβ induced toxicity observed here was also consistent with previous results [32]. CS640 was able to ablate Aβ induced increased tau phosphorylation at Thr181 in mouse primary neurons. Prevention of increased tau phosphorylation in these cultures indicates that this compound can, to some extent, attenuate the development of AD pathology in cell culture. Unfortunately, while reduced tau phosphorylation is promising, it was not paired with improved cell viability. Although inhibition of CaMK1D clearly has an impact on tau phosphorylation levels, alone it was not sufficient to protect the neurons from Aβ induced toxicity. Together, these results suggest that while inhibition of CaMK1D is not sufficient alone to combat AD related neuron death, its effect on tau phosphorylation could contribute to a multi-drug strategy.

As research on inhibitors of CaMK1D and AD continues, such compounds will need to be tested for blood-brain barrier permeability. This may require further optimization. Increased selectivity for CaMK1D over other CaMKI family members to increase efficacy could also be optimized. The effects of these inhibitors on additional phosphorylation sites for tau could be examined, to determine the extent that CaMK1D inhibitors can alter the phosphorylation of tau. Further assays exploring morphological changes to neurons, apoptotic markers, and tau aggregation and localization would provide further clarity to the AD pathway involving CaMK1D. Such future studies will benefit from our results showing that CaMK1D specific inhibitors are not toxic to mouse primary neuron cultures up to 1 µM, and are able to prevent Aβ induced tau phosphorylation at non-toxic concentrations. Although the current compounds are not able to rescue the cells from Aβ induced toxicity when used alone, they may be beneficial when used in combination with other drug molecules.

## 4. Materials and Methods

### 4.1. Mouse Primary Cortical Neuron Cell Culture Preparation

Mouse primary cortical neuron cell cultures were prepared as described previously [33]. In brief, frontal cortices from day 18 prenatal BALB/c mice were dissected out in HBSS (Gibco HBSS with added 0.6 mM L-glutamine, 30 units/mL penicillin, 30 µg/mL streptomycin, 1 mM sodium pyruvate, and 10 mM HEPES pH 8), trypsinized at 37 °C in Gibco TrypLE Express (ThermoFisher Scientific, Grand Island, NY, USA), triturated, and strained into single cells. These were plated on PDL coated 6 well plates (2 mL of media/well, 1.5 × 10^6^ cells/well) or 96 well plates (100 µL of media/well, 5 × 10^4^ cells/well), grown in neurobasal media supplemented with B27 or N2 and kept in a 37 °C and 5% CO_2_ incubator.

### 4.2. Treatment of Mouse Primary Neuron Cell Cultures

Aβ_1–42_ oligomers were prepared as described previously [34]. Peptides were obtained from rPeptide, and these were monomerized by dissolving in HFIP, and HFIP was evaporated to create 0.1 mg monomer films. These were stored at −20 °C until use. When ready for use, DMSO was added to an Aβ concentration of 5 mM. This was then diluted to 1 mM in sterile ddH2O and incubated at 4 °C for 16 h. Oligomerized Aβ and/or CaMK1D inhibitors were diluted in neurobasal media and added to the cell cultures in the desired concentrations.

### 4.3. MTT Assays

**Figure 7:**
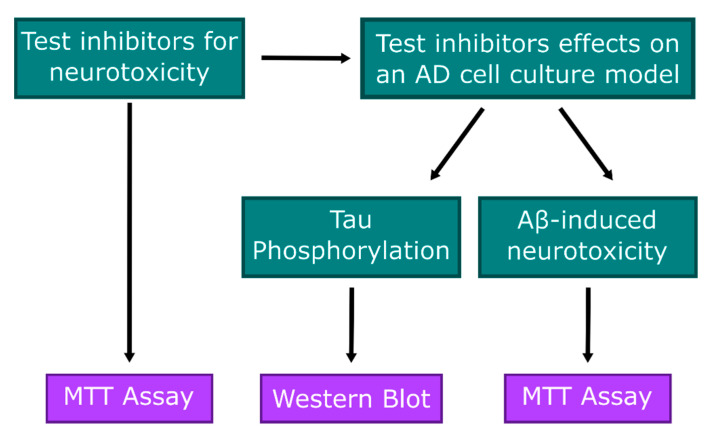
Experimental design. CaMK1D inhibitors were first tested for neurotoxicity by MTT assay. Following these results, the inhibitors were further tested for effects on tau phosphorylation and Aβ-induced neurotoxicity by western blot and MTT assay, respectively.

Primary cells were treated with the desired Aβ_1–42_ oligomers and/or CaMK1D inhibitors (Figure 7). The low MTT signal control was treated with 10% DMSO, and the maximum MTT signal control was treated with only 0.4% DMSO, to match the amount of DMSO in experimental wells. All treatments lasted 24 h. Following these treatments, the media was aspirated, and was replaced with 5 mg/mL MTT reagent from Invitrogen diluted ten times in neurobasal media. The cells were incubated in a 37 °C and 5% CO_2_ incubator for 4–6 h, after which the MTT media was carefully aspirated, leaving the purple formazan crystals, and replaced with 50 µL of DMSO in each well. Plates were shaken gently for 5 min, and absorbance was read at 630 nm and 570 nm. Absorbance readings at 630 nm were subtracted from those at 570 nm, to control for any small changes in turbidity between wells. The viability was then calculated using the following formula:

(1) $$Viability(\%)=\frac{Experimental\;MTT\;Signal-Low\;MTT\;signal}{Maximum\;MTT\;Signal-Low\;MTT\;Signal}\times 100\%$$

### 4.4. LDH Assays

Primary cells were treated with the desired with Aβ_1–42_ oligomers and/or specific CaMK1D inhibitors for 24 h, and LDH assays were performed using Promega’s CytoTox 96^TM^ Non-Radioactive Cytotoxicity Assay (Madison, WI, USA). The low LDH release control cells were treated with only 0.4% DMSO, to match the amount of DMSO in experimental wells, while the maximum LDH release control was treated with 10 µL of 10X Lysis Solution. After these treatments, 50 µL of media were transferred to a fresh 96 well plate, and 50 µL of CytoTox 96^TM^ Reagent was added. The plate was protected from light and incubated at room temperature for 30 min to 1 h. After this, 50 µL of stop solution was added, and absorbance was read at 630 nM and 490 nM. Absorbance readings at 630 nm were subtracted from those at 490 nm, to control for any small changes in turbidity between wells. The cytotoxicity was calculated using the following formula:

(2) $$Cytotoxicity(\%)=\frac{Experimental\;LDH\;Release-Low\;LDH\;Release}{Maximum\;LDH\;Release-Low\;LDH\;Release}\times 100\%$$

### 4.5. Western Blots

After treatment with Aβ and/or CaMK1D inhibitors for 12 h (Figure 7), 6 well plates containing primary neurons were placed on ice, and media was aspirated. Plates were rinsed twice with ice cold PBS, and cells were removed by scraping each well in 500 µL ice cold PBS. Cell suspensions for each plate were centrifuged for 5 min at 500× *g*. Cell pellets were flash frozen in liquid nitrogen, and stored at −80 °C. Once needed, cells were thawed on ice, and lysed by freeze-thaw in RIPA buffer with 1:50 protease inhibitor cocktail (P8340; Sigma-Aldrich, St. Louis, MO, USA), 25 mM NaF, 20 mM sodium pyrophosphate, and 10 mM sodium orthovanadate. Lysates were centrifuged at 21,000× *g* for 1 h, and the supernatant was transferred to a new microcentrifuge tube. Protein concentration of the supernatant was determined by Bio-Rad protein assay (5000006; Hercules, CA, USA). Gradient SDS-PAGE gels (4–20%) from Bio-Rad (17000436; Hercules, CA, USA) were loaded so that the amount of protein in each lane was equal and run at 225 V for 27 min in Tris/Glycine/SDS buffer. Gels were wet transferred to a methanol-activated PVDF membrane at 80 V for 1 h in transfer buffer (25 mM Tris, 192 mM glycine). Membranes were incubated with agitation in blocking buffer (2% fish skin gelatin, 25 mM NaF, 20 mM sodium pyrophosphate, 10 mM sodium orthovanadate, 20 mM Tris pH 7.5, and 150 mM NaCl) for 1–4 h. Primary antibodies were diluted in blocking solution, and incubated with the membranes at 4 °C. The Tg5 antibody, a generous gift from Peter Davies, was diluted 1:500, the AT270 antibody was diluted to 1:250 and the β-Actin antibody was diluted to 1:1000. Membranes were washed once for 10 min, followed by three times for 5 min in Tris-buffered saline with Tween (20 mM Tris pH 7.5, 150 mM NaCl and 0.1% Tween20). Secondary antibodies were diluted 1:3000 in blocking buffer and incubated with membranes for 2–4 h at room temperature. Membranes underwent one 10 min wash, followed by three 5 min washes in TBST, before incubating with ECL substrate for 5 min. Membranes were imaged using a Licor Odyssey^®^ Fc imaging system (Lincoln, NE, USA). Blots were quantified by densitometry using ImageJ.

## Data Availability

Not applicable.

## Sample Availability

Samples of the compounds are available from the corresponding authors of the respective publications.
